# The Transcription Factor *CcMYB330* Regulates Capsaicinoid Biosynthesis in Pepper Fruits

**DOI:** 10.3390/ijms26041438

**Published:** 2025-02-08

**Authors:** Hong Cheng, Mingxian Zhang, Guining Fang, Mengjuan Li, Ruihao Zhang, Qiaoli Xie, Shu Han, Junheng Lv, Minghua Deng

**Affiliations:** 1 Key Laboratory of Vegetable Biology of Yunnan Province, College of Landscape and Horticulture, Yunnan Agricultural University, Kunming 650201, China; 2022210204@stu.ynau.edu.cn (H.C.); zmx11234566@163.com (M.Z.); 2024210211@stu.ynau.edu.cn (G.F.); 13150515440@163.com (M.L.); zrh@yaas.org.cn (R.Z.); yyhs7724@sina.com (S.H.); 2 College of Bioengineering, Chongqing University, Chongqing 400044, China; qiaolixie@cqu.edu.cn

**Keywords:** *Capsicum chinense*, *CcMYB330*, capsaicinoids, regulation

## Abstract

Pepper is an important vegetable and economic crop, and the MYB family is one of the most numerous transcription factor families in plants, extensively participating in various biological processes such as plant growth, development, and stress resistance. In this study, *CcMYB330* is identified as a differentially expressed gene in the pepper fruit, and *CcMYB330* is expressed with higher expression levels in the placenta and pericarp at different development stages of pepper fruit. Analysis of the promoter *cis*-elements revealed that this gene contains not only core elements but also environmental factor response elements and plant hormone response elements. The silencing of *CcMYB330* could reduce the capsaicinoid accumulation in pepper fruit, while the overexpression of *CcMYB330* could increase capsaicinoid accumulation. Additionally, silencing or overexpressing *CcMYB330* could regulate the expression of structural genes involved in capsaicinoid biosynthesis. In addition, through yeast one-hybrid experiments, we identified an interaction between *CcMYB330* and the capsaicinoid biosynthesis structural gene *CcPAL*. Further evidence from EMSA experiments and dual luciferase assays confirmed that *CcMYB330* can bind to the *cis*-element ACCAACAACCAAA in the *CcPAL* promoter. These results indicate that *CcMYB330* may regulate the synthesis of capsaicinoids by modulating structural genes in the capsaicinoid biosynthesis pathway, providing new insights into the regulatory mechanisms of capsaicinoid synthesis.

## 1. Introduction

Pepper is an annual or perennial plant belonging to the *Solanaceae* family, and it is an important vegetable crop. Secondary metabolites in plants are compounds that are not essential for plant growth [1]. Capsaicinoids, a unique type of secondary metabolite found in peppers, can be processed not only as food additives but also play a role in medical, military, and chemical industries [2,3]. Studies have shown that an 8% capsaicin patch can alleviate neuropathic pain [4]; capsaicin can significantly reduce the proliferation of parental and cisplatin-resistant mesothelioma cells [5]; as an active substance in ship anti-fouling systems, capsaicin can reduce risks to the marine environment, offering substantial ecological benefits [6].

**Figure 1:**
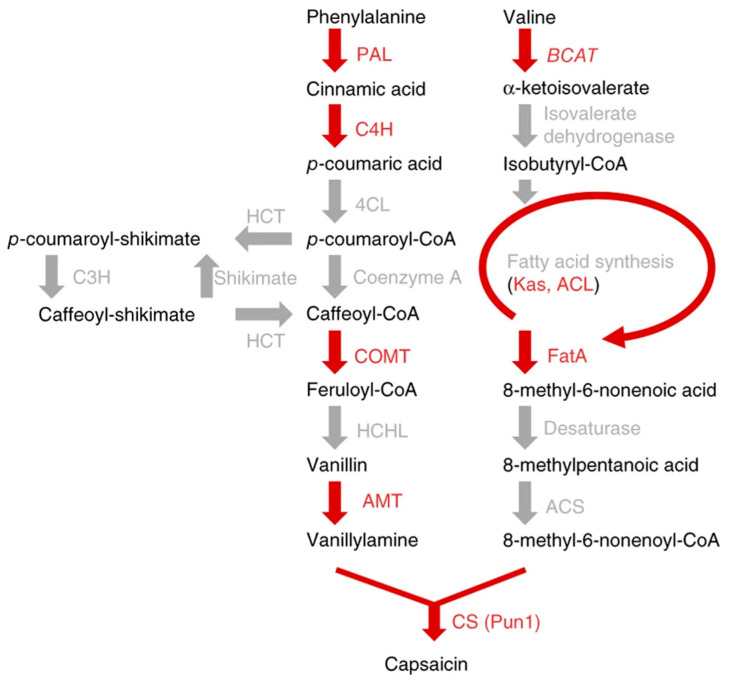
Schematic diagram of the biosynthetic pathway of capsaicin [8].

The biosynthesis pathways of capsaicinoids (Figure 1) primarily include the phenylalanine pathway and the branched-chain fatty acid pathway [7,8]. The phenylalanine pathway converts phenylalanine into vanillylamine through a series of enzyme actions, while the branched-chain fatty acid pathway converts valine into 8-methyl-6-decenoyl-CoA via multiple enzymes. Vanillylamine and 8-methyl-6-decenoyl-CoA are then combined by capsaicin synthase to produce capsaicinoid compounds. The synthesis pathway of capsaicinoids has been extensively studied, and their content is influenced by factors such as the cultivar, growing environment, and fruit developmental stages. Transcription factors play crucial roles in plant growth, stress responses, and gene expression. Studies indicate that transcription factors like *CaMYB31* [9,10], *CaMYB48* [11], *CcERF2* [12], *CaMYB37* [13], and *CcMYB4-12* [14] are related to the biosynthesis of capsaicinoids. Silencing of the *CcERF2* gene leads to the decreased expression of capsaicin synthesis genes and lower capsaicin content [12]; yeast one-hybrid and dual luciferase reporter assays have proven that *CaMYB37* can directly bind to the promoter of the capsaicin biosynthesis structural gene *AT3* and activate its transcription [13], while *CcMYB4-12* can bind to the promoter of *CcPAL2* to inhibit its transcription [14], thereby regulating the biosynthesis of capsaicinoids. The MYB transcription factor family is one of the largest protein families in plants, and MYB transcription factors have been reported to participate in the regulation of secondary metabolite synthesis. *MYB20*, *MYB42*, *MYB43*, and *MYB85* can directly activate the biosynthesis of lignin and phenylalanine; moreover, these MYB proteins can directly activate the transcription repressors of flavonoid biosynthesis [15]. Based on CRISPR/Cas9 gene editing technology, knocking out *DcMYB11c* significantly reduces anthocyanin content. Additionally, *DcMYB11c* can bind directly to the promoters of *DcUCGXT1* and *DcSAT1*, activating the expression of *DcUCG9T1* and *DcSAT1* responsible for anthocyanin glycosylation and acylation, thereby affecting the accumulation of anthocyanin pigments in carrot purple petioles [16]. Silencing the *StMYB168* gene results in reduced phenolic acid content in potato tubers, while silencing the *StMYB24* and *StMYB144* genes leads to decreased lipid derivative content. Overexpressing these genes increases secondary metabolite content. In summary, *StMYB24*, *StMYB144*, and *StMYB168* play certain roles in regulating suberin synthesis [17].

As the hottest pepper in China [18], ‘Shuanla’ (*Capsicum chinense*) is an ideal material for studying capsaicinoid biosynthesis. However, its biosynthetic mechanism remains largely unexplored. Previously, transcriptome analysis showed that the expression level of *CcMYB330* was higher in the late stage of pepper fruit development, which is consistent with the accumulation of capsaicin. It is speculated that it may be a transcription factor involved in capsaicin synthesis. Investigating its role in regulating capsaicinoid synthesis can provide new insights into the regulatory mechanisms of capsaicinoid biosynthesis.

## 2. Results

### 2.1. Cloning and Sequence Analysis of the CcMYB330

**Figure 2:**
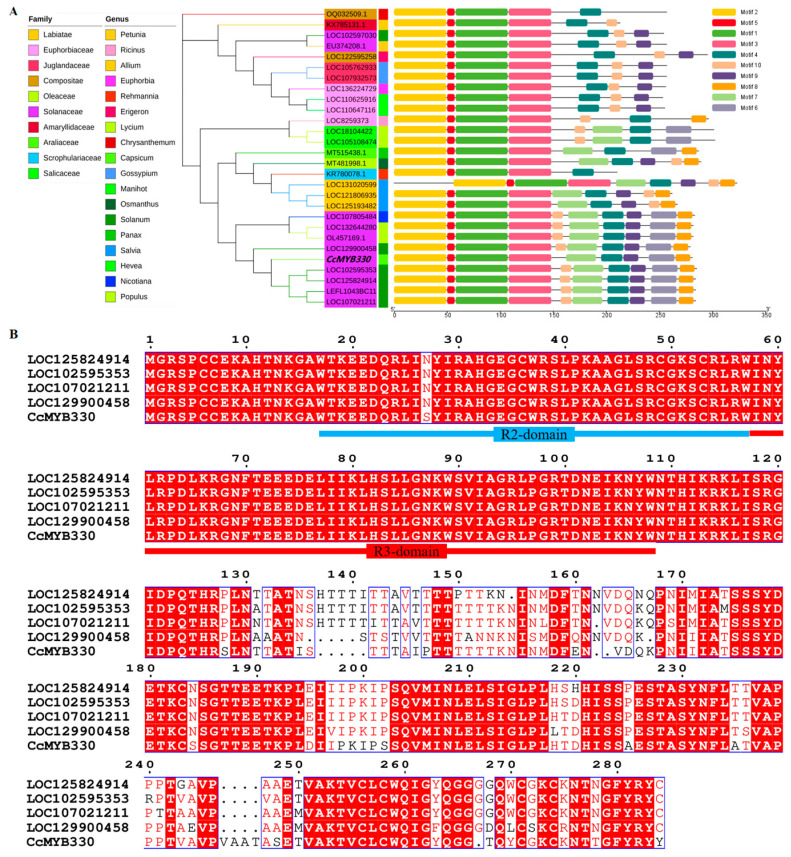
Sequence structure and evolutionary characteristics analysis of *CcMYB330*. (**A**) Phylogenetic tree and promoter *cis*-element analysis of MYB transcription factors. (**B**) Alignment of *MYB330* amino acid sequences.

Using ‘Shuanla’ fruit as material, the *CcMYB330* gene was cloned using its cDNA as a template, with an ORF length of 846 bp. The protein domain was analyzed using NCBI online tools, and the results indicated that the *CcMYB330* transcription factor belongs to the PLN03091 superfamily. A phylogenetic tree was constructed using the NCBI database and MEGA 11 software with the neighbor-joining method. The results showed that within the *Solanaceae* family, the closest relatives are from the *Lamiaceae* family, and the most distant relatives are from the *Asteraceae* family. Within the *Solanaceae*, it is more closely related to the genus *Solanum* and more distantly related to the genus *Nicotiana*. After conducting a significance test on motifs, five different conserved motifs, namely Motif 2, Motif 5, Motif 1, Motif 3, and Motif 4, were identified as common motifs in the predicted plants, which may play an important role in the function of these proteins (Figure 2A). Through multiple sequence alignment, it was found that *MYB330* in five species of the Solanaceae family, including ‘Shuanla’, all contain R2 and R3 domains (Figure 2B). This part of the study provides important evidence for further discussion on protein function.

### 2.2. Characterization of CcMYB330

**Figure 3:**
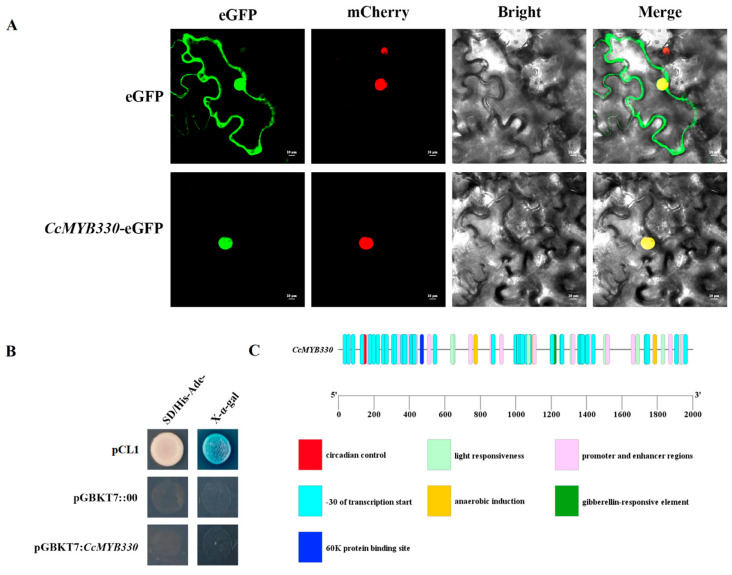
Analysis of characteristics of *CcMYB330*. (**A**) Subcellular localization results of *CcMYB330*. (**B**) *CcMYB330* lacks transcriptional activation activity. pCL1: as a positive control; pGBKT7::00: as a negative control. (**C**) Prediction of *cis*-elements in the *CcMYB330* promoter.

To verify that *CcMYB330* is localized in the nucleus, first, *CcMYB330* was constructed into the pCambia1300 vector and then transiently transformed into tobacco leaves. The results showed that when mCherry and pCambia1300::00 were co-transformed, under two-photon laser scanning microscopy, they were located on the cell membrane and nucleus (Figure 3A), indicating that this system is correct. When mCherry and pCambia1300::*CcMYB330* were co-transformed, the fluorescence distribution of the fusion protein was in the nucleus (Figure 3A), consistent with the predicted results, indicating that the *CcMYB330* protein is localized in the nucleus. To understand the transcriptional activity of *CcMYB330*, a yeast hybrid assay was used for testing. The results showed that pCL1(positive control) grew well on the SD/His-Ade medium and turned blue on the SD/His-Ade medium coated with X-α-gal. Both pGBKT7::00 (negative control) and pGBKT7::*CcMYB330* grew poorly on SD/His-Ade medium and did not turn blue on SD/His-Ade medium coated with X-α-gal (Figure 3B), indicating that *CcMYB330* does not have transcriptional activation activity. Plant CARE predicted that the 2000 bp promoter *cis*-element upstream of the *CcMYB330* gene contains not only core elements, such as promoter and enhancer regions and -30 transcription start sites, but also environmental factor response elements and plant hormone response elements. Among them, there are nine light response elements, one circadian rhythm response element, three anaerobic induction response elements, one gibberellin response element, and one 60K protein binding site response element, indicating that *CcMYB330* responds to hormones and stresses during growth. Additionally, the presence of the 60K protein binding site response element is more conducive to understanding the regulatory mechanism of the *CcMYB330* protein (Figure 3C).

### 2.3. Expression Analysis of CcMYB330

**Figure 4:**
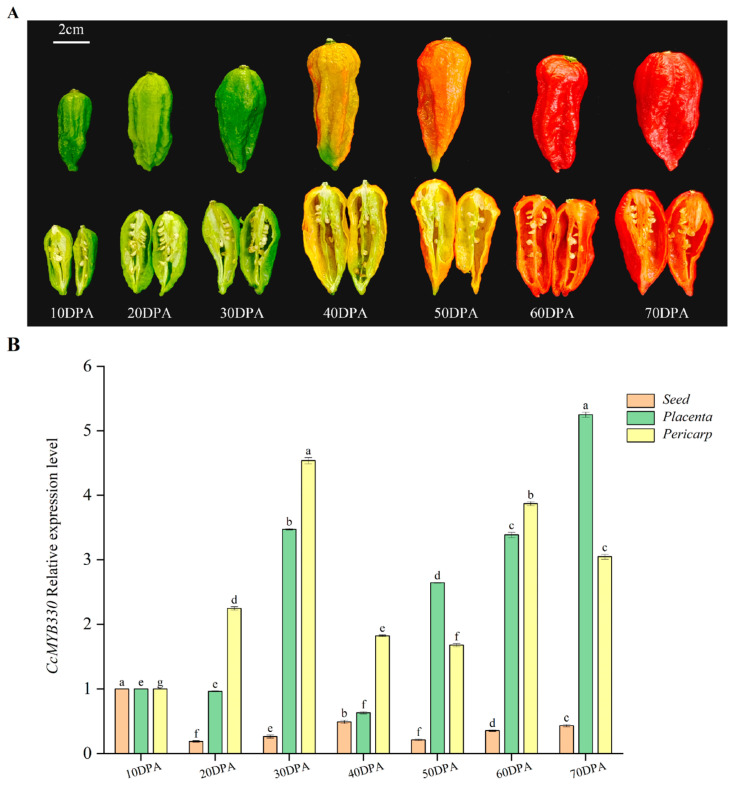
Expression analysis of *CcMYB330*. (**A**) Fruits at different developmental stages. (**B**) Relative expression levels of *CcMYB330* in different tissues of the fruit placenta at various stages. The letters above the bars indicate significant differences determined by Student’s *t*-tests (*p* < 0.05).

Real-time fluorescent PCR technology was used to measure the expression levels of *CcMYB330* in different tissue stages of pepper fruits. The results showed that its expression levels were high in both the pericarp and placenta, with the lowest amount in seeds. Over time, the expression level first increased, decreased slightly, and subsequently showed an increasing trend. Specifically, at 70 days post-anthesis (DPA), the expression level of *CcMYB330* was highest in the placenta, while at 30 DPA, it was highest in the pericarp (Figure 4B).

### 2.4. CcMYB330 Silencing Reduces Capsaicin Accumulation in Pepper Fruits

**Figure 5:**
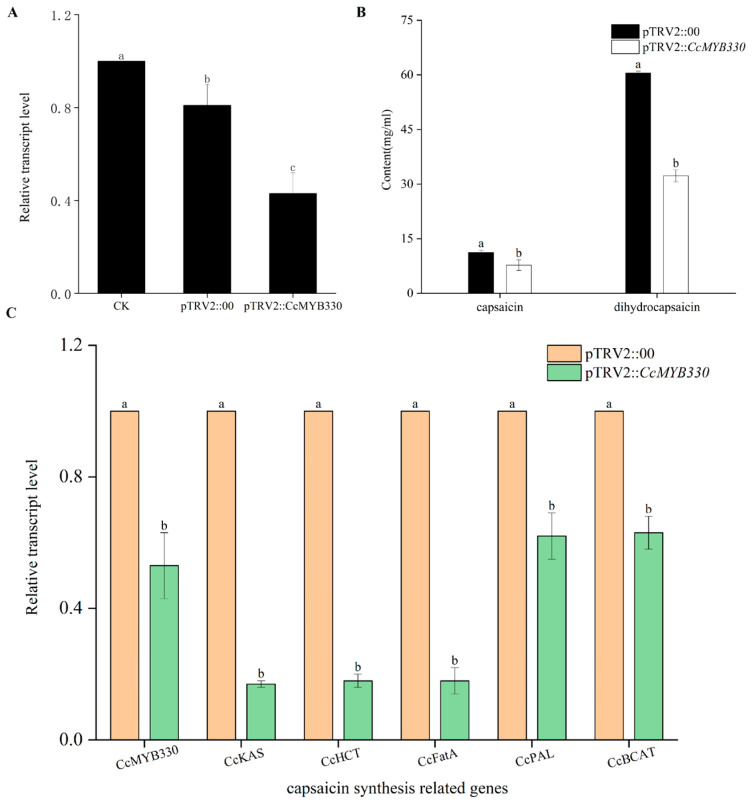
Effects of *CcMYB330* gene silencing. (**A**) Relative expression levels of the *CcMYB330* gene in silenced fruits and the letters above the column indicate significant differences. pTRV2::00: as a negative control. (**B**) Contents of capsaicin and dihydrocapsaicin after silencing of the *CcMYB330* gene. pTRV2::00: as a control group and it represents the transient expression of pTRV1 and the pTRV2 empty vector; pTRV2::*CcMYB330*: as an experimental group and it represents the transient expression of pTRV1 and the pTRV2-*CcMYB330*. (**C**) Relative expression levels of capsaicin biosynthetic genes in the placenta of silenced fruits. The letters above the bars indicate significant differences determined by Student’s *t*-tests (*p* < 0.05).

Virus-induced gene silencing technology was used to infect the placenta of pepper fruits 20 days post-anthesis. Ten days after inoculation, qRT-PCR results showed that compared to pTRV2::00, the expression level of *CcMYB330* in the infected pepper fruit placenta decreased, confirming that the expression of *CcMYB330* was silenced (Figure 5A). To further explore the impact of *CcMYB330* gene silencing on capsaicin accumulation, high-performance liquid chromatography was used to detect the content of capsaicin and dihydrocapsaicin. The results indicated that both capsaicin and dihydrocapsaicin significantly decreased compared to pTRV2::00 (Figure 5B), indicating that *CcMYB330* has a regulatory effect on the synthesis of capsaicin. Additionally, the expression levels of the capsaicin biosynthetic structural genes *CcKAS*, *CcHCT*, *CcFatA*, *CcPAL*, and *CcBCAT* were all downregulated to varying degrees after silencing *CcMYB330*, with the most significant downregulation observed in *CcKAS*, followed by *CcHCT* (Figure 5C).

### 2.5. Transient Overexpression of CcMYB330 Increase Capsaicin Accumulation in Pepper Fruits

**Figure 6:**
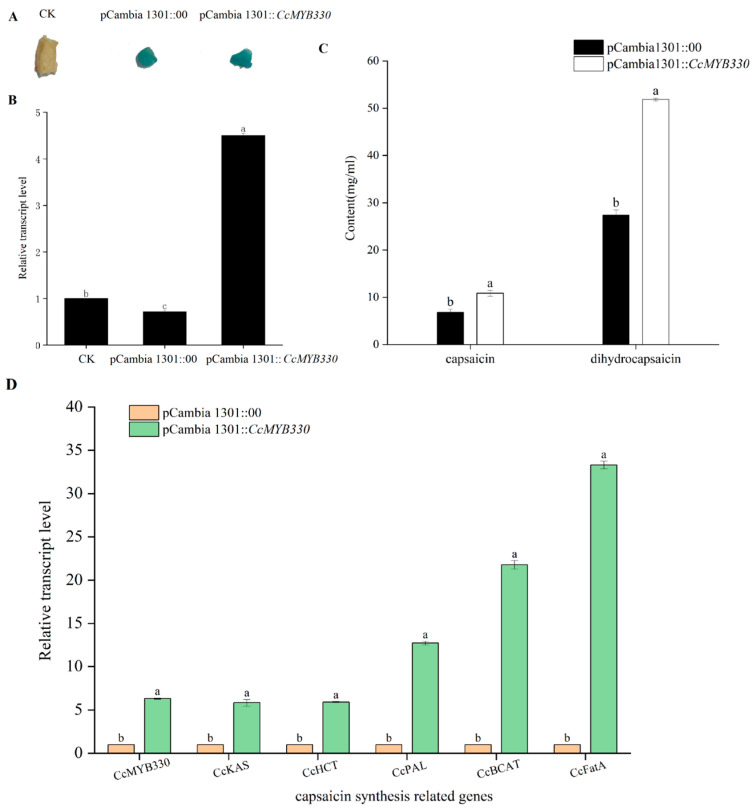
Effects of transient overexpression of the *CcMYB330* gene on capsaicin synthesis. (**A**) GUS staining images of different treatments in the overexpression system. pCambia 1301::00: as a negative control. (**B**) Relative expression levels of the *CcMYB330* gene in silenced fruits. (**C**) Contents of capsaicin and dihydrocapsaicin after transient overexpression of the *CcMYB330* gene. pCambia 1301::00: as a control group and it represents the transient expression of the pCambia 1301 empty vector; pCambia 1301::*CcMYB330*: as an experimental group and it represents the transient expression of the pCambia 1301-*CcMYB330*. (**D**) Relative expression levels of structural genes for capsaicin synthesis in pepper fruits with transient overexpression of the *CcMYB330*. The letters above the bars indicate significant differences determined by Student’s *t*-tests (*p* < 0.05).

Using transient overexpression technology, pepper fruit placenta was infected. After 10 days of infection, the placenta was subjected to GUS staining and subsequently decolorized with 70% ethanol. The results showed that both pCambia1301::00 and pCambia1301::*CcMYB330* were successfully stained (Figure 6A), confirming the successful transient overexpression of *CcMYB330*. qRT-PCR analysis was performed on the placenta 10 days post-infection, indicating that the expression level of *CcMYB330* after transient overexpression was five-fold higher than that of pCambia1301::00 (Figure 6B). Subsequently, high-performance liquid chromatography was used to measure the capsaicin and dihydrocapsaicin content in the positive fruits after the overexpression of *CcMYB330*. The results showed that compared to the control, both capsaicin and dihydrocapsaicin significantly increased (Figure 6C). After the overexpression of *CcMYB330*, the expression levels of the structural genes in the capsaicin synthesis pathway (*CcKAS*, *CcHCT*, *CcPAL*, *CcBCAT*, *CcFatA*) were upregulated to varying degrees, with *CcFatA* increasing by 33-fold (Figure 6D).

### 2.6. CcMYB330 Binding to the CcPAL Promoter

**Figure 7:**
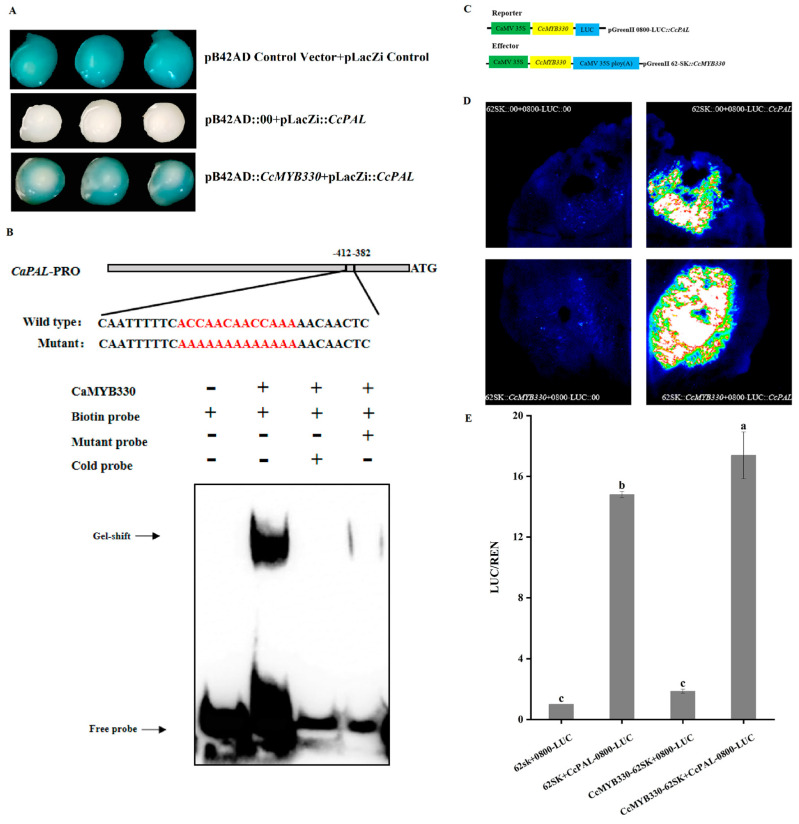
Interaction between *CcMYB330* and the *CcPAL* promoter. (**A**) Yeast one-hybrid experiment demonstrating the interaction between *CcMYB330* and the *CcPAL* promoter. pLacZi Control Vector + pB42AD Control Vector: as a positive control; pLacZi::*CcPAL* + pb42AD::00: as a negative control; pLacZi::*CcPAL* + pb42AD::*CcMYB330*: as an experimental group. (**B**) Electrophoretic mobility shift assay showing that the *CcMYB330* protein can bind to the *CcPAL* promoter element ACCAACAACCAAA. (**C**) Schematic diagram of the dual luciferase reporter gene constructs for 0800-LUC::*CcPAL* and effector gene 62SK::*CcMYB330*. (**D**) LUC in vivo imaging shows that *CcMYB330* activates *CcPAL* transcription. pGreenII 62SK + pGreenII 0800-LUC: as a negative control; pGreenII 62SK::*CcMYB330* + pGreenII 0800-LUC::*CcPAL*:: as an experimental group. (**E**) The ratio of LUC to REN in the LUC assay indicates activity. The letters above the bars indicate significant differences determined by Student’s *t*-tests (*p* < 0.05).

The constructed pB42AD::*CcMYB330* was co-transformed with the pLacZi promoter, followed by plating on SD/-Ura-Trp plates. After 2–4 days of culture, the colonies were harvested and spread on SD/-Ura-Trp + Gal + Raf + X-gal plates. After an additional 2–3 days of incubation, the plates were observed for blue coloration of the colonies. The results indicated that the positive control colonies turned blue, confirming the success of the system. Compared to the colonies co-transformed with pB42AD::00 and pLacZi::*CcPAL*, those co-transformed with pB42AD::*CcMYB330* and pLacZi::*CcPAL* turned blue (Figure 7A), suggesting an interaction between the *CcMYB330* protein and the *CcPAL* promoter.

*CcMYB330* was recombinantly transformed with pGEX-4T-1, followed by protein induction and transformation. The ACCAACAACCAAA element on the *CcPAL* promoter was modified with 5′ Biotin and then subjected to EMSA experiments. The results showed no migration band in the negative control, indicating a successful system. The strongest signal was observed for the complex formed by the labeled DNA fragment alone. When unlabeled DNA fragments were added along with the biotin-labeled DNA fragments, competition between the unlabeled and biotin-labeled DNA fragments led to the disappearance of the complex signal. This indicates that the *CcMYB330* protein can specifically bind to the ACCAACAACCAAA sequence of the *CcPAL* promoter with strong affinity (Figure 7B).

To clarify the regulatory mechanism of *CcMYB330*, a dual luciferase reporter gene assay was conducted. Agrobacterium containing the reporter gene 0800-LUC::*CcPAL* and the effector gene 62SK::*CcMYB330* were co-infiltrated into tobacco leaves (Figure 7C). After 3 days of culture, in vivo imaging was performed. The results showed that fluorescence signals were detected in leaves containing only the reporter gene, but the fluorescence signal was stronger when both effector and reporter genes were present (Figure 7D). The LUC enzyme activity measurement results were consistent with the fluorescence intensity imaging results (Figure 7E). This indicates that *CcMYB330* can directly regulate *CcPAL*, thereby controlling the expression of capsaicin.

## 3. Discussion

Capsaicinoids are a unique class of compounds synthesized in the placenta of peppers, which can prevent animal damage to the fruit and play an important role in food additives and pharmaceutical analgesics [19,20,21]. The MYB family is a large and functionally diverse group of transcription factors that play a certain function in plant physiological processes and secondary metabolism regulation [22,23,24,25,26,27]. MYB transcription factors also regulate the biosynthesis of capsaicin in peppers. Using VIGS technology to silence *CaMYB108*, it was found that the content of capsaicin significantly decreased, while transient overexpression of *CaMYB108* led to an increase in capsaicin content, preliminarily proving that the *CaMYB108* transcription factor can regulate the synthesis of capsaicin [28]. *CcMYB24* may negatively regulate the synthesis of capsaicin by regulating the expression of key genes in the phenylpropanoid metabolism and its branch pathways [29], and *MYB31* can regulate the expression level of capsaicin in chili pepper pericarp [30]. *Cis*-acting elements are sequences that can affect gene expression and serve as binding sites for transcription factors. By subjecting peppers to different shading treatments, it was found that the accumulation of capsaicin differed among varieties, indicating that the accumulation of capsaicin is related to light intensity [31]. Peppers treated with salicylic acid and methyl jasmonate showed increased accumulation of capsaicinoids [32]. By analyzing the promoter of *CcMYB330*, we found that it contains light response elements and gibberellin response elements, suggesting that light and hormones can also influence the synthesis of capsaicin in peppers. Additionally, the promoter predicts one 60K protein binding site response element and yeast hybrid experiments indicate that *CcMYB330* has no self-activation activity, suggesting it may form complexes with other proteins to activate downstream reporter gene expression, thereby regulating the biosynthesis of capsaicin. In roses, both *RcMYB1-RcbLHL42-RcTTG1* and *RcMYB1-RcEGL1-RcTTG* MBW complexes enhance anthocyanin accumulation [33]; in Arabidopsis, *PAP1* and *TT8* synergistically activate *MYBL2* transcription to prevent excessive anthocyanin accumulation, maintaining a balance between strong light adaptation and plant growth [34]. In *T. cinerariifolium*, after MeJA treatment, the expression level of *TcMYB8* was significantly upregulated. In addition, research has found that JAZ4 may interact with *TcMYB8*, indicating that the regulation of pyrethrin synthesis by *TcMYB8* in response to MeJA is related to the jasmonate signaling pathway [35]. The synthesis of capsaicin is a complex mechanism, and the process may be associated with the gibberellin signaling pathway, so our study provides new insights into the synthesis of capsaicin.

The expression level of the *CcMYB330* gene obtained in this experiment shows a trend of increasing first, then decreasing, and then increasing again at different stages of the pepper placenta. Overall, *CcMYB330* has similar expression levels in the pericarp and placenta, with the lowest in the seeds, suggesting that *CcMYB330* has a certain impact on other biosynthetic processes in peppers. Studies have shown that R2R3-MYB transcription factors play an important regulatory role in the biosynthesis of secondary metabolites in plants. In pears, *PbMYB5*-like directly binds to the promoters of *CHI* and *F3H* genes, thereby positively regulating the biosynthesis of phenylalanine-related metabolites [36]. The overexpression of *SbMYB12* significantly promotes the accumulation of baicalin and wogonoside in hairy roots; moreover, it can bind to the promoters of *SbCCL7-4*, *SbCHI-2*, and *SbF6H-1* to activate their expression, indicating that *SbMYB12* positively regulates the production of baicalin and wogonoside [37]. In this study, silencing *CcMYB330* resulted in reduced capsaicinoid content, with varying degrees of downregulation in the expression levels of *CcFatA*, *CcHCT*, *CcKAS*, *CcPAL*, and *CcBCAT* in the capsaicin synthesis pathway, whereas transient overexpression of *CcMYB330* had the opposite effect, suggesting that *CcMYB330* can positively regulate the biosynthesis of capsaicin. Moderate amounts of capsaicinoids can promote plant growth and development, but excessive accumulation of capsaicinoids may reduce plant stress resistance. In grapes, *VvHY5* activates *VvMYBA1*, inducing the expression of *VvUFGT* and leading to an increase in anthocyanin biosynthesis. When the concentration of anthocyanins reaches a certain level, *VvBBX44* expression is activated. Then, *VvBBX44* directly inhibits the expression of *VvMYBA1* and *VvHY5* to prevent excessive accumulation of anthocyanins [38]. Interestingly, in our study, we found that the effect of *CcFatA* seemed to be more obvious when the *CcMYB330* gene was transiently overexpressed or silenced. However, it was found that *CcMYB330* did not interact with this gene in previous experiments, suggesting that *CcMYB330* and other protein formation complexes jointly regulate the synthesis of capsaicin, which laid the foundation for subsequent work.

Studies have shown that MYB can directly regulate gene expression by binding to *cis*-elements. Through yeast one-hybrid assays and EMSA, it was shown that *AhbHLH12*1 can bind to the G/E-box regions of *AhPOD*, *AhCAT* and *AhSOD* promoters to promoting their expression and enhancing the antioxidant enzyme activity of peanuts [39]. *FfMYB15* can recognize the *cis*-element (CAACCA) in the *FfCEL6B* promoter, and the transient expression of *FfMYB15* significantly increased the Luc/Ren ratio of the reporter gene containing *FfCEL6B* through transient dual-luciferase assays, indicating that FfMYB15 can bind to and activate the *FfCEL6B* promoter [40]. The yeast one-hybrid assay and EMSA have demonstrated that *CiMYB42* can bind to the TTGTTG sequence in the *CiOSC* promoter to regulate its expression, thereby regulating the limonoid biosynthesis [41]. This experiment screened a structural gene *CcPAL* interacting with *CcMYB330* in the capsaicin synthesis pathway through yeast one-hybrid experiments; this gene is a key enzyme in the metabolism of phenylpropanoid and the first enzyme in the phenylpropanoid pathway of capsaicin biosynthesis. To explore the interaction mechanism between them, gel shift experiments further proved that *CcMYB3330* can recognize the ACCAACAACCAAA *cis*-element on the *CcPAL* promoter. Although progress has been made in understanding the regulation mechanism of capsaicin biosynthesis, the study of the MYB family’s regulation of capsaicin remains limited. Future research could delve deeper into the regulation of *CcMYB330* on pepper capsaicin biosynthesis, providing a reference for cultivating more high-quality pepper varieties.

## 4. Materials and Methods

### 4.1. Plant Material

The materials used in this study were ‘Shuanla’ fruit and Nicotiana benthamiana. The ‘Shuanla’ was germinated in March 2024 and transplanted to a greenhouse in June for water and fertilizer management and pest and disease control. *N. benthamiana* seeds were sown into plug trays, transplanted to small pots after 15 days, placed in an incubator with 18–22 °C light, and regularly fertilized and watered.

### 4.2. Cloning and Sequence Analysis of CcMYB330

Total RNA was extracted using the plant RNA extraction kit from Beijing Huayueyang Biotechnology Co., Ltd. (Beijing, China) First-strand cDNA synthesis was performed using the reverse transcription kit provided by Nanjing Nuoweizan Biotechnology Co., Ltd. (Nanjing, China). Specific primers were designed using SnapGene 4.1.8 software and synthesized by Beijing Qingke Biotechnology Co., Ltd. (Beijing, China). The pepper cDNA served as the template for cloning *CcMYB330*. The conserved domains of *CcMYB330* were analyzed using the online NCBI database. Phylogenetic trees were constructed using the neighbor-joining method in MEGA 11 with 1000 bootstrap replications based on the NCBI database (https://www.ncbi.nlm.nih.gov/ (accessed on 5 February 2024)). Sequence alignments of *MYB330* from five Solanaceae species including ‘Shuanla’ were conducted using ESPript 3.0 (https://espript.ibcp.fr/ESPript/ESPript/ (accessed on 5 February 2024)). The promoter *cis*-elements of *CcMYB330* were analyzed using PlantCARE (https://bioinformatics.psb.ugent.be/webtools/plantcare/html/ (accessed on 7 February 2024)). All the primer sequences are listed in Table S1.

### 4.3. Subcellular Localization

Primers were designed in SnapGene 4.1.8 software, adding 18–25 bp homologous arms containing Sac I and BamH I restriction sites at the 5′ ends of the upstream and downstream primers. Pepper fruit cDNA was used as the template for cloning and gel recovery. The fragment was then connected to the pCambia1300 vector using Takara’s homologous recombination enzyme. The product was transformed into DH5α cells using the heat shock method provided by Beijing Bomaide Gene Technology Co., Ltd. Single colonies were picked for PCR verification to screen positive monoclonal colonies. After successful verification, the vector was transferred into GV3101 via heat shock, and single clones were selected for PCR validation. Positive colonies were expanded and stored. When tobacco plants reached a suitable size, they were infected. pCambia1300::00, mCherry (a red fluorescent signal located in the nucleus), and pCambia1300::*CcMYB330* were cultured in LB medium containing kanamycin and rifampicin until the OD600 reached 0.8–1.0 at 4000 rpm and then resuspended in infection solution containing MgCl*_2_*, MES, and AS to maintain the OD600 within 0.5–0.8 range. This was incubated at 28 °C in the dark for 3 h. Then, pCambia1300::00 and pCambia1300::*CcMYB330* were mixed with mCherry at a 1:1 ratio to infect tobacco leaves. After infection, the tobacco was kept in a dark incubator at 18 °C for one day and then moved to alternating 16 h light/8 h dark conditions for continued cultivation. Two days later, leaf samples were observed under a two-photon laser scanning microscope (Japan, manufacturer: Nikon, equipment model: A1 MP+). All the primer sequences are listed in Table S1.

### 4.4. Transcriptional Activity Analysis of CcMYB330

The pGBKT7::*CcMYB330* vector was constructed following the method described in Section 4.3. The pGBKT7::00, pGBKT7::*CcMYB330*, and pcl1 vectors were transformed into Y2H yeast strains. pGBKT7::00 and pGBKT7::*CcMYB330* were plated on SD/Trp medium, while pcl1 was plated on SD/Leu medium. After 3–5 days of incubation at 30 °C, the transformed yeast cells were transferred to SD/His-Ade medium with X-α-gal plated on SD/His-Ade medium. Incubation continued for 1–2 days, and growth and color development of the yeast were observed. All the primer sequences are listed in Table S1.

### 4.5. Gene Expression Analysis of CcMYB330

Sampling was conducted on the pericarp, seeds, and placenta of ‘Shuanla’ fruits at 10, 20, 30, 40, 50, 60, and 70 days after flowering. RNA was extracted and reverse transcribed into cDNA following the method in Section 4.2. qRT-PCR primers for the *CcMYB330* gene were designed using the NCBI website. The pericarp, seeds, and placenta from 10 days after flowering served as controls, with CcACTIN as the internal reference gene. The Beijing Lanbolide Biotechnology Co., Ltd. (Beijing, China) fluorescent quantitative kit was used for qRT-PCR with three replicates, and the relative expression level of *CcMYB330* was calculated using the 2^−ΔΔct^ method. All the primer sequences are listed in Table S1.

### 4.6. VIGS Identification of CcMYB330 in Pepper

A 300 bp fragment of *CcMYB330* was selected using the SGN VIGS website (https://vigs.solgenomics.net/ (accessed on 11 February 2023)), and the pepper cDNA was used as a template for cloning and constructing the pTRV2::*CcMYB330* vector. Infection of pepper placenta followed the method described in Section 4.3. Ten days later, sampling was conducted on the pepper placenta. All samples underwent qRT-PCR validation, and positive fruits were also tested for the relative expression levels of structural genes in the capsaicin biosynthesis pathway using qRT-PCR primers designed similarly to those for *CcMYB330*. All the primer sequences are listed in Table S1.

### 4.7. Transient Overexpression Identification of CcMYB330 in Pepper

The pCambia1301::*CcMYB330* transient overexpression vector was constructed following the method in Section 4.3 and used to infect pepper fruits 20 days after flowering. qRT-PCR was used to determine the relative expression levels of *CcMYB330* and structural genes in the capsaicin biosynthesis pathway. Additionally, GUS staining was performed on pepper placenta 10 days post-infection to verify successful infection. All the primer sequences are listed in Table S1.

### 4.8. Extraction and Determination of Capsaicinoids

Capsaicinoids were extracted from pepper placenta according to GB/T21266-2007 [42] with slight modifications. A 0.5 g powder sample of pepper placenta was extracted using an equal volume mixture of methanol and tetrahydrofuran. The supernatant was concentrated using a rotary evaporator and determined by high-performance liquid chromatography with a detection wavelength of 280 nm.

### 4.9. Yeast One-Hybrid Experiment

The pB42AD::*CcMYB330* and pLacZi::*CcPAL* vectors were constructed following the method mentioned in Section 4.3 and co-transformed into yeast cells. After 2–4 days of incubation at 30 °C on SD/-Ura-Trp plates, colonies were collected and streaked onto SD/-Ura-Trp + X-gal plates. After 2–3 days of incubation at 30 °C, the plates were observed for colony color changes. All the primer sequences are listed in Table S1.

### 4.10. Electrophoretic Mobility Shift Assay (EMSA)

The pGEX-4T-1::*CcMYB330* vector was constructed and transformed into *E. coli* strain BL21(DE3). After PCR identification of positive clones, bacterial cultures were induced for expression and verified by agarose gel electrophoresis. Purification and induction were conducted according to the GST purification kit instructions. Primers with binding elements were synthesized by Sangon Biotech (Shanghai) Co., Ltd. (Shanghai, China), with the biotin probe end-labeled with 5′ Biotin for EMSA operations according to the Beyotime EMSA kit (Shanghai, China) instructions. All the primer sequences are listed in Table S1.

### 4.11. Dual Luciferase Assay

Effector genes pGreenII 62-SK and reporter genes pGreenII 08-SK were constructed and transformed into *Agrobacterium tumefaciens* (GV3101) with sequencing confirmation. After sequencing and archiving, the reporter and effector genes were mixed at a 1:9 ratio for injection into tobacco leaves. After 3 days, live imaging and tissue sampling for dual-luciferase assay using the Beyotime Dual-Luciferase Reporter Assay Kit (Shanghai, China) were performed to measure firefly and Renilla luciferase activities. All the primer sequences are listed in Table S1.

### 4.12. Statistical Analysis

All data were analyzed using SPSS 25.0 software for Student’s *t*-tests. These values are expressed as means ± standard deviation. *p* < 0.05 is considered statistically significant.

## 5. Conclusions

In summary, after the silencing or transient overexpression of *CcMYB330*, the structural genes involved in capsaicin biosynthesis were significantly downregulated or upregulated, respectively. Correspondingly, the synthesis and accumulation of capsaicinoids also decreased or increased, indicating that *CcMYB330* may play a positive regulatory role in capsaicin synthesis. Yeast one-hybrid experiments, gel shift assays, and dual-luciferase reporter assays showed that *CcMYB330* can influence capsaicin synthesis by binding to the *cis*-element ACCAACAACCAAA in the *CcPAL* promoter. Additionally, *CcMYB330* may also interact with other transcription factors to jointly regulate capsaicin synthesis, and further research is needed to elucidate the underlying regulatory mechanisms.

## Data Availability

All data supporting the findings of this study are available within the paper and the Supplementary Data published online.

## Supplementary Materials

The following supporting information can be downloaded at https://www.mdpi.com/article/10.3390/ijms26041438/s1.

## References

[1] Yang W., Zhang L., Yang Y., Xiang H., Yang P. (2024). Plant secondary metabolites-mediated plant defense against bacteria and fungi pathogens. Plant Physiol. Biochem. PPB.

[2] Li X., Zhang Y., Zhou C., Li X., Zou X., Ou L., Tao Y. (2024). The changes of rhizosphere microbial communities in pepper varieties with different capsaicinoids. Front. Microbiol..

[3] Orzuna-Orzuna J.F., Godina-Rodríguez J.E., Garay-Martínez J.R., Lara-Bueno A. (2024). Capsaicin as a Dietary Additive for Dairy Cows: A Meta-Analysis on Performance, Milk Composition, Digestibility, Rumen Fermentation, and Serum Metabolites. Animals.

[4] Goodwin B., Mitchell J., Major E., Podwojniak A., Brancaccio H., Rusinak K., King M., Tahir H. (2024). The efficacy of topical 8% capsaicin patches for the treatment of postsurgical neuropathic pain: A systematic review. Pain Manag..

[5] Andretta E., Costa A., Ventura E., Quintiliani M., Damiano S., Giordano A., Morrione A., Ciarcia R. (2024). Capsaicin Exerts Antitumor Activity in Mesothelioma Cells. Nutrients.

[6] Wang J., Shi T., Yang X., Han W., Zhou Y. (2014). Environmental risk assessment on capsaicin used as active substance for antifouling system on ships. Chemosphere.

[7] Milde R., Schnabel A., Ditfe T., Hoehenwarter W., Proksch C., Westermann B., Vogt T. (2022). Chemical Synthesis of Trans 8-Methyl-6-Nonenoyl-CoA and Functional Expression Unravel Capsaicin Synthase Activity Encoded by the Pun1 Locus. Molecules.

[8] Kim S., Park M., Yeom S.-I., Kim Y.-M., Lee J.M., Lee H.-A., Seo E., Choi J., Cheong K., Kim K.-T. (2014). Genome sequence of the hot pepper provides insights into the evolution of pungency in Capsicum species. Nat. Genet..

[9] Han K., Jang S., Lee J.H., Lee D.G., Kwon J.K., Kang B.C. (2019). A MYB transcription factor is a candidate to control pungency in *Capsicum annuum*. TAG Theor. Appl. Genet..

[10] Arce-Rodríguez M.L., Ochoa-Alejo N. (2017). An R2R3-MYB Transcription Factor Regulates Capsaicinoid Biosynthesis. Plant Physiol..

[11] Sun B., Zhou X., Chen C., Chen C., Chen K., Chen M., Liu S., Chen G., Cao B., Cao F. (2020). Coexpression network analysis reveals an MYB transcriptional activator involved in capsaicinoid biosynthesis in hot peppers. Hortic. Res..

[12] Wen J., Lv J., Zhao K., Zhang X., Li Z., Zhang H., Huo J., Wan H., Wang Z., Zhu H. (2022). Ethylene-Inducible AP2/ERF Transcription Factor Involved in the Capsaicinoid Biosynthesis in *Capsicum*. Front. Plant Sci..

[13] Liu Y., Zhang Z., Fang K., Shan Q., He L., Dai X., Zou X., Liu F. (2022). Genome-Wide Analysis of the MYB-Related Transcription Factor Family in Pepper and Functional Studies of *CaMYB37* Involvement in Capsaicin Biosynthesis. Int. J. Mol. Sci..

[14] Yu S., Zhang W., Zhang L., Wu D., Fu G., Yang M., Wu K., Wu Z., Deng Q., Zhu J. (2024). Negative regulation of CcPAL2 gene expression by the repressor transcription factor CcMYB4-12 modulates lignin and capsaicin biosynthesis in *Capsicum chinense* fruits. Int. J. Biol. Macromol..

[15] Geng P., Zhang S., Liu J., Zhao C., Wu J., Cao Y., Fu C., Han X., He H., Zhao Q. (2019). *MYB20*, *MYB42*, *MYB43*, and *MYB85* Regulate Phenylalanine and Lignin Biosynthesis during Secondary Cell Wall Formation. Plant Physiol..

[16] Duan A.Q., Deng Y.J., Tan S.S., Xu Z.S., Xiong A.S. (2023). A MYB activator, DcMYB11c, regulates carrot anthocyanins accumulation in petiole but not taproot. Plant Cell Environ..

[17] Han Y., Yang R., Zhang X., Wang Q., Wang Y., Li Y., Prusky D., Bi Y. (2024). *MYB24*, *MYB144*, and *MYB168* positively regulate suberin biosynthesis at potato tuber wounds during healing. Plant J..

[18] Deng M., Wen J., Zhu H., Zou X. (2009). The hottest pepper variety in China. Genet. Resour. Crop Evol..

[19] Das S., Priyadarshani N., Basak P., Maitra P., Bhattacharya S., Bhattacharya S.S. (2023). Capsaicin derived from endemic chili landraces combats *Shigella* pathogen: Insights on intracellular inhibition mechanism. Microb. Pathogenesis..

[20] Mahmood S., Mei T.S., Yee W.X., Hilles A.R., Alelwani W., Bannunah A.M. (2021). Synthesis of Capsaicin Loaded Silver Nanoparticles Using Green Approach and Its Anti-Bacterial Activity Against Human Pathogens. J. Biomed. Nanotechnol..

[21] Chabaane Y., Arce C.M., Glauser G., Benrey B. (2021). Altered capsaicin levels in domesticated chili pepper varieties affect the interaction between a generalist herbivore and its ectoparasitoid. J. Pest Sci..

[22] Wang X., Niu Y., Zheng Y. (2021). Multiple Functions of MYB Transcription Factors in Abiotic Stress Responses. Int. J. Mol. Sci..

[23] Wang Z., Peng Z., Khan S., Qayyum A., Rehman A., Du X. (2024). Unveiling the power of MYB transcription factors: Master regulators of multi-stress responses and development in cotton. Int. J. Biol. Macromol..

[24] An C., Sheng L., Du X., Wang Y., Zhang Y., Song A., Jiang J., Guan Z., Fang W., Chen F. (2019). Overexpression of CmMYB15 provides chrysanthemum resistance to aphids by regulating the biosynthesis of lignin. Hortic. Res..

[25] Niyitanga S., He Q., Li H., Wei H., Ishimwe C., Qi J., Fang P., Xu J., Tao A., Zhang L. (2023). Genome-wide identification and expression analysis of MYB transcription factors involved in fiber formation in white jute (*Corchorus capsularis*). Ind. Crops Prod..

[26] Luo Y., Xu X., Yang L., Zhu X., Du Y., Fang Z. (2024). A R2R3-MYB transcription factor, FeR2R3-MYB, positively regulates anthocyanin biosynthesis and drought tolerance in common buckwheat (*Fagopyrum esculentum*). Plant Physiol. Biochem..

[27] Ambawat S., Sharma P., Yadav N.R., Yadav R.C. (2013). MYB transcription factor genes as regulators for plant responses: An overview. Physiol. Mol. Biol. Plants.

[28] Sun B., Zhu Z., Chen C., Chen G., Cao B., Chen C., Lei J. (2019). Jasmonate-Inducible R2R3-MYB Transcription Factor Regulates Capsaicinoid Biosynthesis and Stamen Development in *Capsicum*. J. Agric. Food Chem..

[29] Yu S., Zhang W., Zhang L., Wu D., Sun P., Huang C., Fu G., Deng Q., Wang Z., Cheng S. (2023). MYB24 Negatively Regulates the Biosynthesis of Lignin and Capsaicin by Affecting the Expression of Key Genes in the Phenylpropanoid Metabolism Pathway in *Capsicum chinense*. Molecules.

[30] Sun B., Chen C., Song J., Zheng P., Wang J., Wei J., Cai W., Chen S., Cai Y., Yuan Y. (2022). The *Capsicum* MYB31 regulates capsaicinoid biosynthesis in the pepper pericarp. Plant Physiol. Biochem. PPB.

[31] Jeeatid N., Suriharn B., Bosland P.W., Techawongstien S. (2017). Light intensity affects capsaicinoid accumulation in hot pepper (*Capsicum chinense* Jacq.) cultivars. Hortic. Environ. Biotechnol..

[32] Ancona-Escalante W.d.R., Baas-Espinola F.M., Castro-Concha L.A., Vázquez-Flota F.A., Zamudio-Maya M., de Miranda-Ham M.L. (2013). Induction of capsaicinoid accumulation in placental tissues of *Capsicum chinense* Jacq. requires primary ammonia assimilation. Plant Cell Tissue Organ Cult. (PCTOC).

[33] He G., Zhang R., Jiang S., Wang H., Ming F. (2023). The MYB transcription factor RcMYB1 plays a central role in rose anthocyanin biosynthesis. Hortic. Res..

[34] Xing M., Xin P., Wang Y., Han C., Lei C., Huang W., Zhang Y., Zhang X., Cheng K., Zhang X. (2024). A negative feedback regulatory module comprising R3-MYB repressor MYBL2 and R2R3-MYB activator PAP1 fine-tunes high light-induced anthocyanin biosynthesis in *Arabidopsis*. J. Exp. Bot..

[35] Zhou L., Li J., Zeng T., Xu Z., Luo J., Zheng R., Wang Y., Wang C. (2022). TcMYB8, a R3-MYB Transcription Factor, Positively Regulates Pyrethrin Biosynthesis in *Tanacetum cinerariifolium*. Int. J. Mol. Sci..

[36] Li S., Zhou Z., Yang Y., Zhou X., Lei D., He R., Zhang Y., Zhang J., Lin Y., Wang Y. (2024). R2R3-MYB transcription factor PbMYB5-like positively regulates the biosynthesis of phenylalanine-related metabolites in pear (*Pyrus bretschneideri*). J. Agric. Food Res..

[37] Wang W., Hu S., Yang J., Zhang C., Zhang T., Wang D., Cao X., Wang Z. (2022). A Novel R2R3-MYB Transcription Factor SbMYB12 Positively Regulates Baicalin Biosynthesis in *Scutellaria baicalensis* Georgi. Int. J. Mol. Sci..

[38] Liu W., Mu H., Yuan L., Li Y., Li Y., Li S., Ren C., Duan W., Fan P., Dai Z. (2023). VvBBX44 and VvMYBA1 form a regulatory feedback loop to balance anthocyanin biosynthesis in grape. Hortic. Res..

[39] Zhao X., Wang Q., Yan C., Sun Q., Wang J., Li C., Yuan C., Mou Y., Shan S. (2023). The bHLH transcription factor AhbHLH121 improves salt tolerance in peanut. Int. J. Bio. Macromol..

[40] Liu Z., Deng B., Yuan H., Zhang B., Liu J., Meng J., Chang M. (2022). Transcription factor FfMYB15 regulates the expression of cellulase gene FfCEL6B during mycelial growth of *Flammulina filiformis*. Microb. Cell Factories.

[41] Zhang P., Liu X., Yu X., Wang F., Long J., Shen W., Jiang D., Zhao X. (2020). The MYB transcription factor CiMYB42 regulates limonoids biosynthesis in citrus. BMC Plant Biol..

[42] (2007). Determination of Total Capsaicinoid Content and Representation of Pungency Degree in Capsicum and Its Products.

