# Supplementary material for: The Transcription Factor CcMYB330 Regulates Capsaicinoid Biosynthesis in Pepper Fruits
Source: Int J Mol Sci. 2025 Feb 8;26(4):1438. doi: 10.3390/ijms26041438 (PMC11854957; doi:10.3390/ijms26041438)
Supplement: Supplementary file 1 [file ijms-26-01438-s001.zip › ijms-3448654-supplementary.pdf]

Table S1: Primers used in this study.

| Primer name | Primer sequences | |
|---|---|---|
| CcMYB330-F1 | ATGGGACGTTCACCTTGT | *CcMYB330* clone |
| CcMYB330-R1 | TCAGTAATATCTGTAAAATCCAGTG | |
| CcMYB330-F2 | AAGACTGCCGGGAAGAACTG | *CcMYB330* expression |
| CcMYB330-R2 | TAGTCGCGGTAGTGTTGAGT | |
| CcMYB330-F3 | TAAGGTTACCGAATTCACATTAAGCGAAAACTCATCAGCC | *CcMYB330* VIGS |
| CcMYB330-R3 | GCTCGGTACCGGATCTGGTAACCCTATTGATAGTTCAAGA | |
| CcMYB330-F4 | AGAACACGGGGGACTCTTGACCATGGTAATGGGACGTTCACCTTGT | *CcMYB330* transient overexpression |
| CcMYB330-R4 | AAAACTAGAAATTTACCCTCAGATCTACGTAATATCTGTAAAATCCAGTGGTG | |
| CcMYB330-F5 | CTCTCGAGCTTTCGCGAGCTCATGGGACGTTCACCTTGT | *CcMYB330 subcellular localization* |
| CcMYB330-R5 | CCCTTGCTCACCATGGATCCGTAATATCTGTAAAATCCAGTGGTGT | |
| CcMYB330-F6 | TCAGAGGAGGACCTGCATATGATGGGACGTTCACCTTGT | *CcMYB330* yeast hybridization |
| CcMYB330-R6 | GCTGCAGGTCGACGGATCCTCAGTAATATCTGTAAAATCCAGTG | |
| CcMYB330-F7 | CCGCGTGGATCCCCGGAATTCATGGGACGTTCACCTTGT | *CcMYB330* EMSA |
| CcMYB330-R7 | GATGCGGCCGCTCGAGTCGACTCAGTAATATCTGTAAAATCCAGTG | |
| ACTIN-F | GCCTAGAAATTTGAGCCTCATT | *ACTIN* expression |
| ACTIN-R | AAGATGGTATTGTTGCTGATGG | |
| CcKAS-F | CTTGGTGATGCAGCAGAAGC | *CcKAS* expression |
| CcKAS-R | AGGCGCGATGTCATGATGTA | |
| CcHCT-F | ACGGCGTCCATCTTACCAAG | *CcHCT* expression |
| CcHCT-R | GGCTAGTGATTGGAGGCACTT | |
| CcPAL-F1 | GTGGCAACCCTTCAATTCCC | *CcPAL* expression |
| CcPAL-R1 | GTCAATCTCCTCACCGGGC | |
| CcBCAT-F | CAGCCTGCTATTTACAGTAGTGA | *CcBCAT* expression |
| CcBCAT-R | ACGATCAAGTTGGCCTTGTC | |
| CcFatA-F | AGGACTTGTGCCACGAAGAG | *CcFatA* expression |
| CcFatA-R1 | TCATGCTGGCATTCACGTCT | |
| CcMYB330-F8 | GATTATGCCTCTCCCGAATTCATGGGACGTTCACCTTGT | *CcMYB330* Y1H |
| CcMYB330-R8 | AGAAGTCCAAAGCTTCTCGAGTCAGTAATATCTGTAAAATCCAGTG | |
| CcPAL-F2 | TTTGATATTGGATCGGAATTCGAGATTTCAC | *CcPAL* Y1H |

| | | |
|---|---|---|
| | CTAATACGTAGGG | |
| CcPAL-R2 | ATACAGAGCACATGCCTCGAGCAATCACTACTAGCTTTCACACC | |
| CcMYB330-F9 | GCTCTAGAACTAGTGGATCCATGGGACGTTCACCTTGT | *CcMYB330* Dual-LUC |
| CcMYB330-R9 | ATCGATAAGCTTGATATCGAATTCTCAGTAATATCTGTAAAATCCAGTG | |
| CcPAL-F3 | TCACTATAGGGCGAATTGGGTACCCGAATGAACTTATTGCACTACTACA | *CcPAL* Dual-LUC |
| CcPAL-R3 | CCGCTCTAGAACTAGTGGATCCTGGATCAAGAAATGGCTAGAGT | |
